# Prognostic Factors in Patients with Rhabdomyosarcoma Using Competing-Risks Analysis: A Study of Cases in the SEER Database

**DOI:** 10.1155/2020/2635486

**Published:** 2020-09-17

**Authors:** Didi Han, Chengzhuo Li, Xiang Li, Qiao Huang, Fengshuo Xu, Shuai Zheng, Hui Wang, Jun Lyu

**Affiliations:** ^1^Department of Clinical Research, The First Affiliated Hospital of Jinan University, Guangzhou, Guangdong Province 510630, China; ^2^School of Public Health, Xi'an Jiaotong University Health Science Center, Xi'an, Shaanxi Province 710061, China; ^3^School of Mechanical Engineering, Xi'an Jiaotong University, Xi'an 710049, China; ^4^Center for Evidence-Based and Translational Medicine, Zhongnan Hospital of Wuhan University, Wuhan 430071, China; ^5^School of Public Health, Shannxi University of Chinese Medicine, Xianyang, Shaanxi, China

## Abstract

**Background:**

Rhabdomyosarcoma (RMS) is a rare malignant soft-tissue sarcoma characterized by a poor outcome and unclear prognostic factors. This study applied a competing-risks analysis using data from the Surveillance, Epidemiology, and End Results (SEER) database to RMS patients, with the aim of identifying more accurate prognostic factors.

**Methods:**

Data of all patients with RMS during 1986–2015 were extracted from the SEER database. We used the competing-risks approach to calculate the cumulative incidence function (CIF) for death due to rhabdomyosarcoma (DTR) and death from other causes (DOC) at each time point. The Fine–Gray subdistribution proportional-hazards model was then applied in univariate and multivariate analyses to determine how the CIF differs between groups and to identify independent prognostic factors. The potential prognostic factors were analyzed using the competing-risks analysis methods in SAS and R statistical software.

**Results:**

This study included 3399 patients with RMS. The 5-year cumulative incidence rates of DTR and DOC after an RMS diagnosis were 39.9% and 8.7%, respectively. The multivariate analysis indicated that age, year of diagnosis, race, primary site, historic stage, tumor size, histology subtype, and surgery status significantly affected the probability of DTR and were independent prognostic factors in patients with RMS. A nomogram model was constructed based on multivariate models for DTR and DOC. The performances of the two models were validated by calibration and discrimination, with C-index values of 0.758 and 0.670, respectively.

**Conclusions:**

A prognostic nomogram model based on the competing-risks model has been established for predicting the probability of death in patients with RMS. This validated prognostic model may be useful when choosing treatment strategies and for predicting survival.

## 1. Introduction

Rhabdomyosarcoma (RMS) involves a rare malignant neoplasm of striated muscle. It constitutes 3% of all soft-tissue sarcomas in adults, while the estimated annual incidence in the US is 4.5 cases per 1 million children and adolescents [1, 2]. RMS is characterized by a poor prognosis and unclear prognostic factors. The primary treatment of RMS includes chemotherapy, surgery, and radiation. The identification of prognostic factors for RMS may help with optimizing treatment protocols [3]. For all soft-tissue sarcomas, RMS accounts for 19% of such cases with adults and 45% of those in children [4]. Rhabdomyosarcoma is derived from primary mesenchymal cells, which presents as the skeletal muscle differentiation. There are two major histologic subtypes of RMS: embryonal (ERMS) for the younger patients and alveolar (ARMS) for older patients. The previous one typically arises in the head/neck and GU locations, while the latter one typically develops in the trunk and extremity locations [5]. There have been many reports on prognostic factors for RMS of the head, neck, limbs, and urogenital system [2, 3, 6–12]. Previous studies [13] have analyzed prognostic factors using the Kaplan–Meier method and the Cox proportional-hazards model, in which outcome events were classified into two categories: death or censored observation. Traditional survival analysis treats such competing risks by censoring, which will lead to an inaccurate survival function [14]. This is because the Kaplan–Meier method and the Cox method treat other competing events as censored, and the resulting estimates might be high or even inconsistent with the data, and this is also called competing-risks bias [15]. Therefore, competing-risks methods based on the subdistribution proportional hazards are recommended. However, to our knowledge, a competing-risks analysis and nomogram for RMS based on the Fine–Gray subdistribution proportional-hazards model have not been reported previously.

This study considered two types of failure event: death due to rhabdomyosarcoma (DTR) and death from other causes (DOC). We conducted a competing-risks analysis of patients with RMS using the Surveillance, Epidemiology, and End Results (SEER) database. We also constructed a simple competing-risks nomogram to evaluate the probabilities of DTR and DOC.

## 2. Patients and Methods

### 2.1. Study Patients

The study sample was derived from the SEER program, which collects demographic, diagnostic, and treatment information on all newly diagnosed cancer patients residing within specific geographic regions of the US. The SEER program collects information on incidence, prevalence, and survival, and its registry currently covers about 28% of the US population. The characteristics of the SEER population are comparable with those of the general US population.

**Figure 1:**
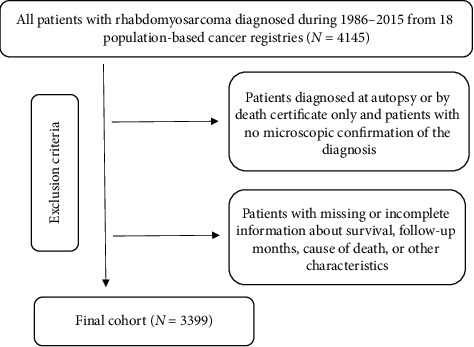
The inclusion and exclusion process of the study sample.

The population included in this study comprised all patients who were diagnosed with RMS between 1986 and 2015, as identified using SEER^∗^Stat software (version 8.3.6.1). The study sample consisted of patients with the following ICD-O-3 (third revision of the International Classification of Diseases for Oncology) histology codes: 8900/0, 8900/3, 8901/3, 8902/3, 8903/0, 8904/0, 8905/0, 8910/3, 8912/3, 8920/3, 8921/3, and 8991/3. Only patients diagnosed with their first primary malignant tumor were included in this study. The following exclusion criteria were applied: (1) diagnosed at autopsy or by death certificate only, (2) no microscopic confirmation of the diagnosis, or (3) missing or incomplete information on survival, follow-up duration, or cause of death. After applying the exclusion criteria, the study population comprised 3399 patients with RMS. The flow chart for data selection is shown in Figure 1.

### 2.2. Defining Patient Characteristics

The following 14 variables related to each RMS case were selected from the SEER database: age, year of diagnosis, race, sex, marital status, primary site, historic stage, tumor size, laterality, histology subtype (HS), radiation sequence with surgery (RS), surgery status, chemotherapy status, and radiotherapy status. Follow-up information and the cause of death were also extracted from the SEER database. Age was classified into <19 and ≥19 years. The year of diagnosis was classified into 1986–1995, 1996–2005, and 2006–2015. Race was classified into white, black, and others. Sex was classified into male and female. Marital status was classified into married, unmarried, and divorced/separated/widowed (DSW). Tumor site was categorized into favorable, unfavorable, and unknown (missing), in accordance with the criteria used for staging pediatric tumors [16]. The head and neck (nonparameningeal), genitourinary (not bladder or prostate), and bile-duct regions were defined as favorable sites, while all other sites were regarded as unfavorable. We used the SEER “Historic Stage A” to classify the tumor stage into localized, regional, and distant. The tumor size was classified into <5, 5–10, ≥10 cm, and “other status.” Laterality was classified into left, right, and “not a paired site.” HS was classified into RMS, embryonal, alveolar, pleomorphic, and other. RS was classified into no radiation and/or cancer-directed surgery (NRS), radiation before and after surgery (WRS), and intraoperative radiation with radiation before or after surgery (IRS). The surgery status and radiotherapy status were defined as receiving or not receiving the corresponding therapy, while the chemotherapy status was classified into receiving and not/unknown.

DTR was the primary endpoint. Consistent with the COD to site code, we classified the endpoint as alive, DTR or DOC. DTR were defined as cases in the SEER database where “SEER cause-specific death classification” was recorded as “Dead (attributable to this cancer dx),” while DOC were defined where this variable was recorded as “Death of other cause.”

### 2.3. Statistical Analysis

The subdistribution proportional-hazards function is defined as the instantaneous possibility of occurrence of a given event in patients who have not experienced that type of event. The cumulative incidence function (CIF) describes the cumulative probability of the occurrence of a given event while accounting for competing events [17]. The Fine–Gray subdistribution proportional-hazards model was, then, applied in univariate and multivariate analyses to determine how the CIF differs between groups and to identify independent prognostic factors. Hazard ratios and their associated 95% confidence intervals were calculated.

The model was internally validated. The C-index was used to measure the discrimination performance of the model and ranged from 0.5 (representing random chance) to 1.0 (representing a perfectly discriminating model). The model calibration—referring to the agreement between predicted and observed outcomes—was also checked. Furthermore, Fine–Gray proportional-hazards regression was performed to predict the 1-, 3-, and 5-year probabilities of the two competing death outcomes (i.e., DTR and DOC).

All statistical analyses were performed using SAS (version 9.4, SPSS, Chicago, IL, USA) and R (version 3.6.1; https://www.r-project.org/) statistical software. Several R packages were used to construct the model (*survival*, *cmprsk*, *rms*, *mstate*, *survism*, *statmod*, and *eha*), while the *pec* and *risk regression* packages were used to evaluate the model performance. All statistical tests were two-sided, with *P* < 0.05 considered to be indicative of statistical significance.

## 3. Results

### 3.1. Patient Characteristics

The study included 3399 patients who met the inclusion criteria, of whom 1335 (39.28%) were DTR patients and 344 (10.12%) were DOC patients. The 3399 patients included 1976 (58.1%) who were aged <19 years and 1489 (43.8%) males. Most of the patients were white (*n* = 2535, 74.6%), unmarried (72.6%), and had RMS at unfavorable sites (67.1%). Based on the historic stage, 1127 (48.7%) of patients had localized tumors, 24.5% had regional RMS, and 26.8% had distant metastasis. Tumors of size 5–10 cm were the most common (*n* = 1977, 51.5%), and most of the tumors (*n* = 1890) were on the right side. Around 18.1% of patients were diagnosed with RMS: 38.9% with embryonal RMS, 24.8% with alveolar RMS, 10.4% with pleomorphic RMS, and 7.8% with other RMS. NRS, WRS, and IRS were applied to 65.1%, 34.3%, and 0.6% of the patients, respectively. Surgery, chemotherapy, and radiotherapy had been applied to 62.6%, 83.8%, and 56.7% of the patients, respectively. The DTR patients comprised 341 married and 877 unmarried cases, while for the DOC group, the marital distribution was 137 (married) and 155 (unmarried), respectively. The proportions of those with primary-site surgery were 50.9% (*n* = 680) and 67.7% (*n* = 233) related to the DTR and DOC patients, respectively. For radiotherapy, 54.0% of DTR patients (*n* = 721) were treated with the radiation, while 40.4% (*n* = 139) of DOC patients were given the same therapy. Chemotherapy was applied to 83.7% (*n* = 1118) of the DTR patients and 51.5% (*n* = 177) of the DOC patients. The demographic and tumor characteristics are listed in Table 1.

### 3.2. Univariate Analysis of the Prognosis of Rhabdomyosarcoma

**Figure 2:**
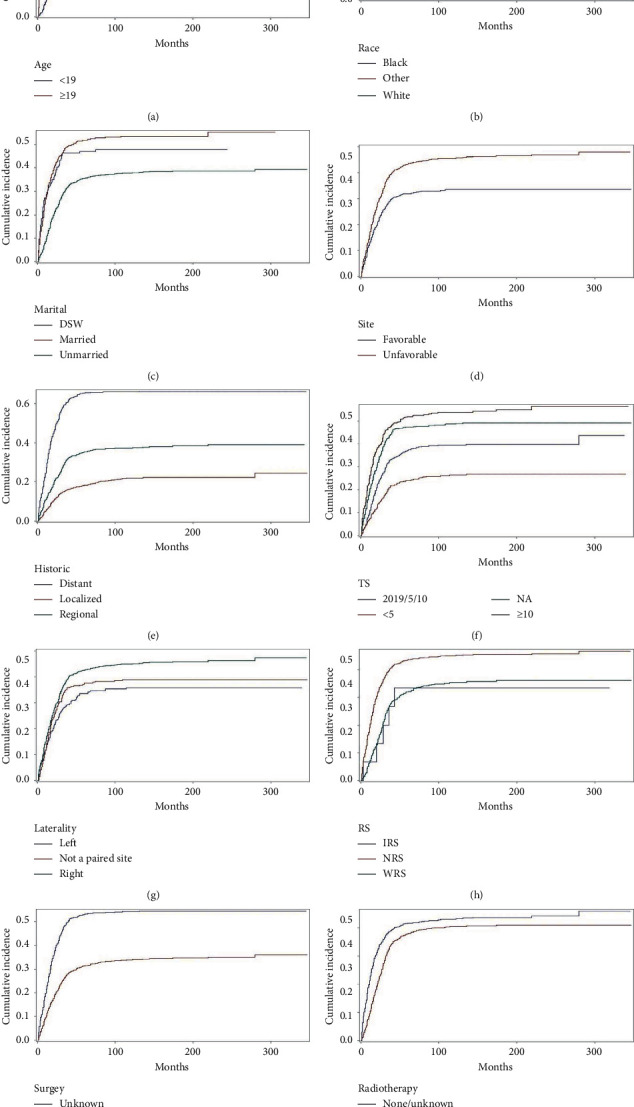
Cumulative incidence curves of cause-specific death according to patient characteristics (a–j).

The CIFs of DTR and DOC were 15.7% and 4.7%, respectively, at 1 year, 35.3% and 7.4% at 3 years, and 39.9% and 8.7% at 5 years. The 1-, 3-, and 5-year estimates of the cumulative incidence rates of DTR and DOC according to age, year of diagnosis, race, sex, marital status, primary site, historic stage, tumor size, laterality, HS, RS, surgery status, chemotherapy status, and radiotherapy status are presented in Table 2. The analysis of 14 variables by univariate Gray's test revealed the characteristics for an age <19 years, white race, unmarried status, favorable site, localized historic stage, a tumor size <5 cm, and left laterality. The embryonal RMS was associated with a lower probability of DTR, while in NRS, no surgery, chemotherapy, and radiotherapy were associated with a higher probability of DTR. The historic stage and surgery status were not significantly related to the cumulative incidence of DTR. Age, year, race, sex, marital, site, historic stage, tumor size, laterality, HS, RS, chemotherapy, and radiotherapy significantly refer to the cumulative incidence of competing mortality. The CIF curves of DTR are shown in Figures 2(a)–2(j).

### 3.3. Multivariate Analysis of the Prognosis of Rhabdomyosarcoma

Table 3 presents the results of a multivariate analysis, which was performed by a Fine–Gray subdistribution proportional-hazards model. After adjusting for the variables that were significant in the univariate analysis by the CIF, the multivariate analysis found that age, race, primary site, historic stage, tumor size, HS, and surgery status could significantly affect DTR in patients with RMS. The probability of DTR was higher in RMS patients with an advanced age (sdHR = 1.915, *P* < 0.001), unfavorable site (sdHR = 1.241, *P*=0.011), and larger tumor (sdHR = 1.447, *P* < 0.001). Patients who belong to white race (sdHR = 0.837, *P*=0.042), localized historic stage (sdHR = 0.294, *P* < 0.001), embryonal RMS (sdHR = 0.617, *P* < 0.001), and received surgery (sdHR = 0.720, *P*=0.002) owned a lower probability of DTR. However, the tumor-related factors of the year of diagnosis, race, sex, primary site, historic stage, tumor size, laterality, HS, RS, surgery status, and radiotherapy status were no longer associated with DOC. Instead, only age, marital status, and chemotherapy status were significantly associated with DOC, advanced age and DSW. In addition, no chemotherapy involves a higher risk of DOC.

### 3.4. Construction and Validation of the Nomogram

**Figure 3:**
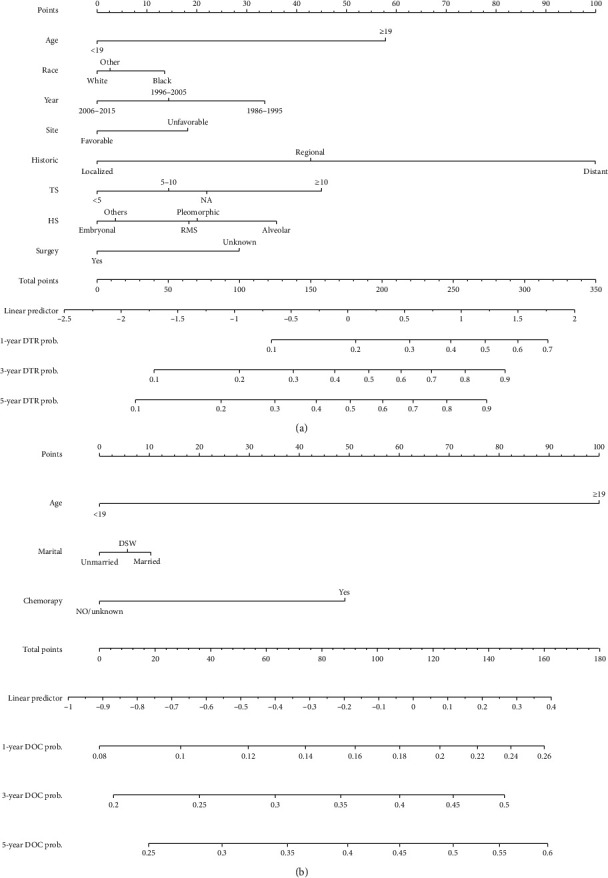
Competing-risks nomogram predicting 1-year, 3-year, and 5-year cumulative probabilities for DTR and DOC in patients with RMS. (a) RMS cancer-specific death; (b) other cause-specific death; TS, tumor size; HS, histology subtype; SEER, Surveillance, Epidemiology, and End Results.

The nomogram that we developed based on the subdistribution proportional-hazards model is shown in Figure 3. All of the independent predictors of DTR and DOC in the entire study population were included in the predictive nomogram established for the 1-, 3-, and 5-year probabilities of DTR and DOC in the training cohort. The discrimination performance of the Fine–Gray model was evaluated based on the C-index, whose values for the 1-, 3-, and 5-year probabilities of DTR for the nomogram were 0.758, 0.714, and 0.707 in the derivation cohort and 0.769, 0.739, and 0.735 in the validation cohort, respectively. The related values of the DOC nomogram were 0.670, 0.620, and 0.609 in the derivation cohort and 0.643, 0.608, and 0.601 in the validation one, respectively.

**Figure 4:**
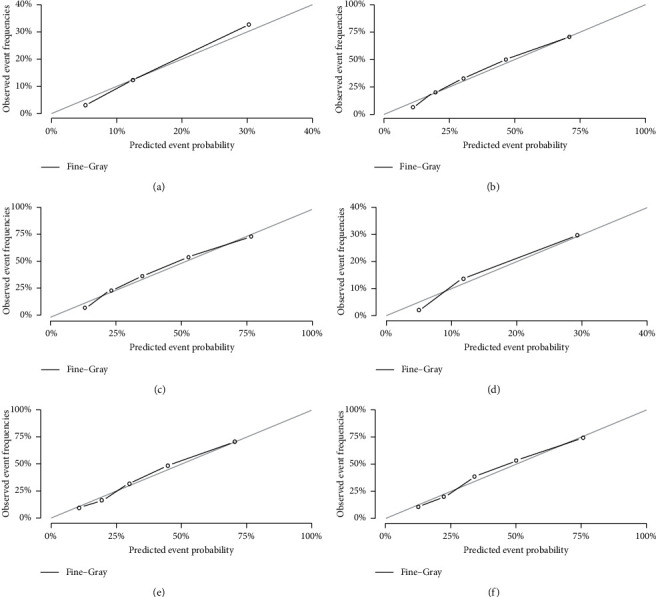
Calibration plots of the nomogram for 1-, 3-, and 5-year predicting RMS cause-specific mortality of the training set (a, b, c) and validation set (d, e, f).

**Figure 5:**
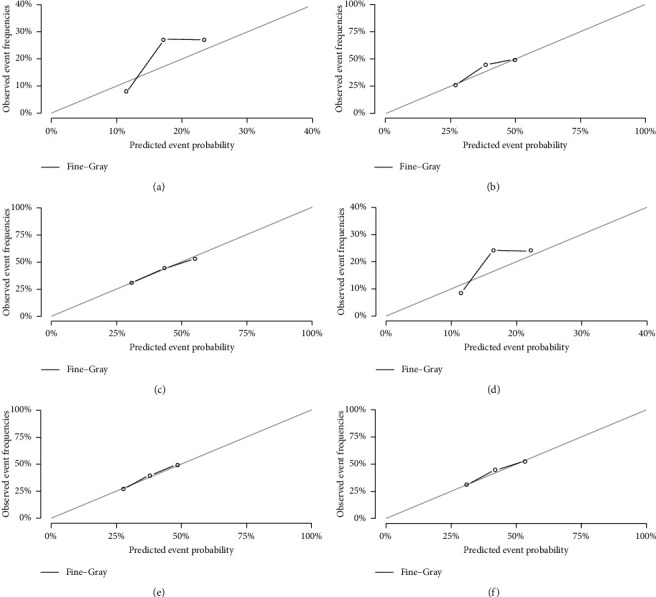
Calibration plots of the nomogram for 1-, 3-, and 5-year predicting other cause-specific mortality of the training set (a, b, c) and validation set (d, e, f).

The internal calibration plots revealed a strong correlation between the predictions estimated by the nomogram and actual observations for both the training and validation cohorts. The calibration plots for 1-, 3-, and 5-year probabilities of DTR are shown in Figures 4 and 5. The dots in the plots fall close to the 45° diagonal line, which suggests that the model was well calibrated for all predictions. The calibration plot showed good agreement between predicted and observed outcomes.

## 4. Discussion

RMS accounts for half of the soft-tissue sarcomas in children. Although it is the most common soft-tissue tumor, it is still rare, accounting for only 3-4% of pediatric cancers [18]. The present study evaluated DTR for 3399 patients with RMS who had been enrolled in the SEER database between 1986 and 2015; calculated the 1-, 3-, and 5-year CIFs; and constructed a nomogram to predict 1-, 3-, and 5-year probabilities of DTR and DOC. We found that 344 of 1699 patients were DOC, comprising 20% of the deaths, and this was taken as censored data based on the common method of survival analysis. The traditional method of analyzing specific causes of death may overestimate the cumulative incidence of each variable. Therefore, the Fine–Gray subdistribution proportional-hazards model was applied in this study to estimate the effects of covariates on the CIF and, therefore, identify the independent prognostic factors for RMS. A concise nomogram based on a competing-risks model was constructed to predict the probabilities of DTR and DOC. This prognostic tool will be useful for determining prognoses and guiding treatment selection.

Previous studies of the prognosis status of RMS have widely used Kaplan–Meier estimates of survival curves and Cox regression models to describe survival trends and identify important prognostic factors [19, 20]. Competing-risks nomograms have been established for other tumors, such as breast cancer, prostate cancer, thyroid cancer, kidney cancer, sarcoma, melanoma, and head and neck squamous cell carcinoma [21–27]. To our knowledge, the present study is the first to construct a nomogram for patients with RMS based on the Fine–Gray subdistribution proportional-hazards model. The model was found to perform well, and the predictive tool is also easy to use because the variables incorporated in the model can be obtained from clinical investigations.

Our univariate CIF analysis showed that the 5-year mortality probabilities for DTR and DOC were 39.5% and 8.7%, respectively. This study is the first to conduct a risk analysis of RMS patients using a cumulative risk model in a competing-risks model and, thereby, identify more accurate prognostic factors. After adjusting for prognostic factors distinguished by the CIF, the Fine–Gray subdistribution proportional-hazards model indicated that the *P* value was statistically significant for age, race, primary site, historic stage, tumor size, HS, and surgery status. The probability of DTR was higher in RMS patients with advanced age, black race, unfavorable site, distant historic stage, larger tumor, alveolar RMS, and no surgery. Similarly, advanced age, DSW, and no chemotherapy increased the probability of DOC. We, then, utilized the independent predictive factors to create a monogram for the 1-, 3-, and 5-year probabilities of DTR and DOC. The C-index values and the calibration curves indicated that the model performed well in both the training and validation cohort.

Variations in survival rates with the age at diagnosis have been observed in many studies [2, 6, 10, 28]. Sultan et al. [16] studied 2600 patients with a diagnosis of RMS and found that the outcomes were consistently worse for adults than for children regardless of the clinical characteristics. Our results were also similar to the previous research [2]; in that, older patients had a higher risk of DTR. In a multivariate analysis, a diagnosis during 1986–1995 was a significant independent predictor of a poor prognosis, which is consistent with previous research. A previous study [10] of survival data divided into decades found a similar trend, with a significantly worse 5-year survival rate during the 1970s (46%) than during the 1980s and 1990s (60% and 64%, respectively). Our analyses of racial differences in RMS incidence and survival were exploratory only due to the smallness of the samples in many of the compared categories.

The tumor primary site was classified into favorable and unfavorable based on criteria used for staging pediatric tumors. Our multivariate analysis results for DTR revealed significant differences. Other studies have also found that the survival rate of RMS is worse at unfavorable sites [16, 29], which is consistent with the present findings. Many authors have concluded that local and regional control is the most important factor for improving long-term survival [30–32]. The results obtained in our multivariate analysis of DTR also indicated that the postoperative survival was better for localized historic stage and regional RMS than for distant RMS, which is consistent with previous findings of distant RMS having an unfavorable prognosis. For smaller tumors with no evidence of metastasis, surgical extirpation alone might be the definitive treatment [33–35]. Moreover, Unsal et al. and Dantonello et al. reported that RMS with tumor size is a risk factor for poor survival [6, 36]. This is consistent with our study finding that a larger tumor was an adverse prognostic factor in DTR patients.

In our study, the survival was better in embryonal than alveolar RMS patients, with 5-year mortality rates of 26.7% and 53.3%, respectively. This finding of alveolar RMS being a significant independent predictor of a poor prognosis is consistent with previous research that found HS to be associated with survival [37]. In a previous study, Dasgupta et al. [5] reported that local surgery treatment is one of the key aspects and the main prognostic factor in treatment of RMS. Moreover, our multivariate analysis results for DTR show that surgery produced significant effects, which is consistent with previous results.

DOC means death due to causes other than RMS, including cardiovascular disease, respiratory disease, and diabetes mellitus. We found that advanced age, DSW, and no chemotherapy increased the probability of DOC. Older age is associated with significant declines in bodily functions and resulting in worse compensatory capabilities. Thus, advanced age is the predominant factor affecting DOC. Being married is associated with a comfortable, confident, and enjoyable emotional state, and married patients also receive social support from their family and have favorable family financial circumstances [38]. Multiagent chemotherapy is currently the indicated treatment for all patients with RMS. Neoadjuvant chemotherapy is also utilized to obtain cytoreduction of unresectable tumors and facilitate subsequent surgical excision [39]. Studies have shown that pediatric RMS has better sensitivity to chemotherapy than those diagnosed in adults [40].

The main strength of this study was that our nomogram can be used to quantify the probability of DTR after a diagnosis of RMS on an individual basis. To our knowledge, this is the first effort to construct a nomogram based on the Fine–Gray subdistribution proportional-hazards model for predicting DTR in RMS. This predictive tool is easy to use because the variables incorporated in the model can be obtained from clinical investigations. Therefore, based on combining clinical features and clinical information, this graphical predictive tool can be easily used by clinicians to make a prognosis judgment in a patient within seconds by drawing a few lines and without requiring any difficult calculations. Furthermore, SEER data are of high quality and are collected in a uniform manner with uniform data standards. The quality control of the SEER program ensures that there are relatively low rates of errors in the cancer registry.

This study was subject to a few limitations. First, there would have been differences from 1986 to 2015 in the types of surgery performed, in the experience of the surgeons, and in the grade classification of tumors, which may have interfered with the results. Second, the SEER database does not provide data on adjuvant therapy, comorbidities, or recurrence rates. Finally, despite being a user-friendly tool for helping clinicians to make clinical decisions, our nomogram did not include all possible prognostic factors and will not always provide an accurate prognosis for individual patients in clinical practice. Therefore, independent external validation is necessary to confirm the efficacy of the model.

## 5. Conclusions

In conclusion, we have evaluated the CIFs of DTR and DOC and performed a competing-risks analysis in patients with RMS using a large population-based sample from the SEER database. We also discovered independent predictive factors of DTR and DOC to build a nomogram. To our knowledge, we have produced the first competing-risks nomogram for calculating the 1-, 3-, and 5-year probabilities of DTR and DOC. Our nomogram performed relatively accurate and allows for objective and accurate selection of the patient population at high risk for rhabdomyosarcoma cause-specific mortality. We believe that these nomograms could be easily used by clinicians to predict prognosis and help determine a personalized treatment for DTR patients. However, further external validation is still needed.

## Figures and Tables

**Table 1:** Baseline characteristics of patients with rhabdomyosarcoma.

Variables	Classification	*N*	Cause-specific death (%)	Death due to other causes (%)
*Total*		3399	1335	344
*Age*	<19	1976 (58.1)	629 (47.1)	57 (16.6)
	≥19	1423 (41.9)	706 (52.9)	287 (83.4)
*Year*	1986–1995	396 (11.7)	168 (12.6)	38 (11.0)
	1996–2005	1250 (36.8)	527 (39.5)	130 (37.8)
	2006–2015	1753 (51.5)	640 (47.9)	176 (51.2)
*Race*	White	2535 (74.6)	961 (72.0)	276 (80.3)
	Black	580 (17.1)	249 (18.7)	51 (14.8)
	Other	284 (8.3)	125 (9.3)	17 (4.9)
*Sex*	Male	1489 (43.8)	591 (44.3)	170 (49.4)
	Female	1910 (56.2)	744 (55.7)	174 (50.6)
*Marital*	Married	682 (20.1)	341 (25.5)	137 (39.8)
	Unmarried	2466 (72.6)	877 (65.7)	115 (33.5)
	DSW	251 (7.3)	117 (8.8)	92 (26.7)
*Site*	Favorable	1118 (32.9)	350 (26.2)	102 (29.7)
	Unfavorable	2281 (67.1)	985 (73.8)	242 (70.3)
*Historic stage*	Localized	1127 (48.7)	224 (16.8)	113 (32.8)
	Regional	1169 (24.5)	409 (30.6)	116 (33.7)
	Distant	1103 (26.8)	702 (52.6)	115 (33.5)
*Tumor size (cm)*	<5	868 (25.1)	199 (14.9)	73 (21.2)
	5–10	969 (51.5)	360 (26.9)	86 (25.0)
	≥10	748 (23.4)	388 (29.1)	94 (27.3)
	Other	814 (23.9)	388 (29.1)	91 (26.5)
*Laterality*	Left	776 (22.8)	270 (20.2)	75 (21.8)
	Right	1890 (55.6)	257 (19.3)	62 (18.0)
	Not a paired site	733 (21.6)	808 (60.5)	207 (60.2)
*HS*	RMS	615 (18.1)	293 (21.9)	125 (36.3)
	Embryonal	1321 (38.9)	354 (26.5)	52 (15.1)
	Alveolar	843 (24.8)	451 (33.8)	53 (15.4)
	Pleomorphic	352 (10.4)	154 (11.5)	91 (26.5)
	Others	268 (7.8)	83 (6.2)	23 (6.7)
*RS*	NRS	2213 (65.1)	947 (70.9)	249 (72.4)
	WRS	1167 (34.3)	382 (28.6)	94 (27.3)
	IRS	19 (0.6)	6 (0.5)	1 (0.3)
*Surgery*	Yes	2127 (62.6)	680 (50.9)	233 (67.7)
	No/unknown	1272 (37.4)	655 (49.1)	111 (32.3)
*Chemotherapy*	Yes	1926 (56.7)	1118 (83.7)	177 (51.5)
	None/unknown	1473 (43.3)	217 (16.3)	167 (48.5)
*Radiotherapy*	Yes	2847 (83.8)	721 (54.0)	139 (40.4)
	No/unknown	552 (16.2)	614 (46.0)	205 (59.6)

DSW: divorced and separated and widowed; RMS: rhabdomyosarcoma; HS: histology subtype; RS: radiation sequence with surgery; NRS: no radiation and/or cancer-directed surgery; WRS: radiation prior to surgery, radiation after surgery, and radiation before and after surgery.

**Table 2:** Univariate analysis of prognostic factors in patients with rhabdomyosarcoma.

Variables	Classification	Cancer-specific mortality (%)			*P*	Non-cancer-specific mortality (%)			*P*
		1 year	3 years	5 years		1 year	3 years	5 years	
*Total*		15.7	35.3	39.5	<0.001	4.7	7.4	8.7	<0.001
*Age*	<19	8.3	26.3	31.3	<0.001	0.5	1.4	2.0	<0.001
	≥19	26.1	47.9	51.1		10.7	16.0	18.2	
*Year*	1986–1995	16.4	34.9	39.7	0.967	2.5	4.8	5.8	0.001
	1996–2005	15.7	35.8	39.6		4.7	6.6	7.4	
	2006–2015	15.6	34.9	39.4		5.2	8.8	10.5	
*Race*	White	15.1	33.8	37.9	0.022	5.0	8.0	9.2	0.022
	Black	17.7	40.0	43.3		4.7	6.7	8.0	
	Other	17.3	38.3	45.5		2.8	4.8	5.7	
*Sex*	Male	17.1	35.9	40.6	0.269	6.3	9.2	10.3	0.011
	Female	14.6	34.7	38.6		3.5	6.1	7.4	
*Marital*	Married	27.1	48.1	51.1	<0.001	10.3	16.8	19.3	<0.001
	Unmarried	11.0	30.6	35.5		1.5	2.7	3.5	
	DSW	31.7	46.8	47.7		21.6	29.3	31.8	
*Site*	Favorable	12.9	28.7	32.0	<0.001	4.0	6.4	7.1	<0.001
	Unfavorable	17.1	38.5	43.2		5.1	7.9	9.5	
*Historic stage*	Localized	5.8	16.1	19.1	0.000	2.8	5.8	7.9	0.841
	Regional	11.8	30.1	34.7		4.7	7.0	8.1	
	Distant	30.0	60.0	65.2		6.7	9.6	10.1	
*Tumor size*	<5	6.6	19.7	22.4	0.000	2.1	4.9	6.5	0.013
	5–10	11.6	33.2	38.0		4.1	6.2	7.5	
	≥10	26.2	47.4	51.9		7.4	10.3	11.6	
	Other	20.8	43.0	47.7		5.9	9.0	9.7	
*Laterality*	Not a paired site	14.6	34.6	38.1	<0.001	4.5	7.2	8.2	<0.001
	Left	12.9	29.1	34.3		3.4	5.5	7.2	
	Right	17.4	38.0	42.1		5.4	8.3	9.5	
*HS*	RMS	26.7	44.8	48.4	0.000	10.3	15.5	18.2	0.000
	Embryonal	9.4	23.1	26.7		1.5	2.3	2.5	
	Alveolar	14.7	47.0	53.3		2.3	4.6	5.9	
	Pleomorphic	26.2	43.4	45.4		14.0	20.4	23.1	
	Other	11.3	24.3	29.0		3.8	6.6	7.5	
*RS*	NRS	20.2	39.7	43.3	<0.001	5.9	8.9	10.0	<0.001
	WRS	7.5	26.9	32.3		2.6	4.8	6.3	
	IRS	5.6	27.8	33.3		0.0	0.0	0.0	
*Surgery*	Yes	11.7	27.2	31.6	0.000	4.5	7.4	9.0	0.053
	No/unknown	22.5	48.7	52.7		5.1	7.6	8.2	
*Chemotherapy*	Yes	14.2	34.8	39.4	0.172	2.4	4.5	5.4	0.000
	No/unknown	23.7	37.6	39.9		16.8	22.5	26.0	
*Radiotherapy*	Yes	10.5	32.6	37.6	<0.001	2.7	4.8	5.7	<0.001
	None/unknown	22.6	38.7	41.9		7.5	10.9	12.5	

DSW: divorced and separated and widowed; RMS: rhabdomyosarcoma; HS: histology subtype; RS: radiation sequence with surgery; NRS: no radiation and/or cancer-directed surgery; WRS: radiation prior to surgery, radiation after surgery, and radiation before and after surgery; IRS: intraoperative radiation and intraoperative rad with other rad before/after surgery.

**Table 3:** Proportional subdistribution hazards models for rhabdomyosarcoma.

Variables	Classification	DTR		*P*	DOC		*P*
		Coefficient	sdHR (95% CI)		Coefficient	sdHR (95% CI)	
*Age*	<19	Reference			Reference		
	≥19	0.650	1.915 (1.586–2.314)	<0.001	1.091	2.978 (1.879–4.720)	<0.001
*Year*	1986–1995	Reference			Reference		
	1996–2005	−0.260	0.771 (0.625–0.950)	0.015	−0.156	0.856 (0.570–1.284)	0.452
	2006–2015	−0.430	0.650 (0.527–0.803)	<0.001	−0.170	0.844 (0.572–1.244)	0.391
*Race*	Black	Reference			Reference		
	White	−0.178	0.837 (0.705–0.993)	0.042	0.069	1.071 (0.734–1.563)	0.722
	Other	−0.138	0.871 (0.663–1.145)	0.322	−0.704	0.495 (0.232–1.056)	0.069
*Sex*	Female	Reference			Reference		
	Male	−0.046	0.955 (0.832–1.097)	0.517	0.184	1.202 (0.918–1.573)	0.180
*Marital status*	DSW	Reference			Reference		
	Married	0.010	1.010 (0.747–1.367)	0.947	−0.565	0.569 (0.406–0.795)	0.001
	Unmarried	−0.142	0.868 (0.629–1.198)	0.388	−0.963	0.382 (0.249–0.585)	<0.001
*Site*	Favorable	Reference			Reference		
	Unfavorable	0.216	1.241 (1.050–1.467)	0.011	0.313	1.367 (0.996–1.877)	0.053
*Historic stage*	Distant	Reference			Reference		
	Localized	−1.225	0.294 (0.239–0.362)	<0.001	−0.215	0.806 (0.546–1.191)	0.280
	Regional	−0.695	0.499 (0.425–0.585)	<0.001	−0.063	0.939 (0.657–1.343)	0.732
*Tumor size*	5–10	Reference			Reference		
	<5	−0.170	0.843 (0.681–1.044)	0.118	−0.189	0.827 (0.561–1.221)	0.340
	≥10	0.370	1.447 (1.199–1.747)	<0.001	−0.228	0.796 (0.545–1.163)	0.238
	Other	0.074	1.077 (0.894–1.298)	0.435	−0.112	0.894 (0.612–1.306)	0.563
*Laterality*	Left	Reference			Reference		
	Not a paired site	0.067	1.070 (0.865–1.323)	0.535	0.202	1.223 (0.811–1.846)	0.337
	Right	0.120	1.128 (0.941–1.351)	0.192	0.316	1.371 (0.948–1.982)	0.094
*HS*	Alveolar	Reference			Reference		
	RMS	−0.253	0.777 (0.625–0.965)	0.022	0.384	1.468 (0.954–2.258)	0.081
	Embryonal	−0.483	0.617 (0.517–0.736)	<0.001	−0.375	0.687 (0.418–1.130)	0.139
	Pleomorphic	−0.208	0.813 (0.606–1.090)	0.166	0.266	1.305 (0.794–2.145)	0.294
	Other	−0.470	0.625 (0.459–0.852)	0.003	0.067	1.069 (0.554–2.063)	0.842
*RS*	IRS	Reference			Reference		
	NRS	0.447	1.564 (0.586–4.176)	0.372	−0.012	0.988 (0.157–6.205)	0.989
	WRS	0.419	1.520 (0.584–3.954)	0.391	0.803	1.255 (0.211–7.457)	0.803
*Surgery*	No/unknown	Reference			Reference		
	Yes	−0.328	0.720 (0.587–0.884)	0.002	−0.195	0.823 (0.545–1.244)	0.356
*Chemotherapy*	No/unknown	Reference			Reference		
	Yes	0.200	1.222 (0.959–1.557)	0.105	−0.792	0.453 (0.328–0.626)	<0.001
*Radiotherapy*	None/unknown	Reference			Reference		
	Yes	−0.148	0.862 (0.700–1.062)	0.163	−0.459	0.632 (0.389–1.026)	0.064

DSW: divorced and separated and widowed; RMS: rhabdomyosarcoma; HS: histology subtype; RS: radiation sequence with surgery; NRS: no radiation and/or cancer-directed surgery; WRS: radiation prior to surgery, radiation after surgery, and radiation before and after surgery; IRS: intraoperative radiation and intraoperative rad with other rad before/after surgery.

## Data Availability

The data used to support the findings of this study are available from the Surveillance, Epidemiology, and End Results (SEER) program (http://ssp://seerstat.imsweb.com: 2038-10025-Nov2019). All authors have access to data from this database. The interpretation and reporting of such data is the sole responsibility of the author.
